# 4BNT162b2 mRNA COVID‐19 vaccine and semen: What do we know?

**DOI:** 10.1111/andr.13199

**Published:** 2022-06-08

**Authors:** Soraya Olana, Rossella Mazzilli, Gerardo Salerno, Virginia Zamponi, Maria Grazia Tarsitano, Maurizio Simmaco, Donatella Paoli, Antongiulio Faggiano

**Affiliations:** ^1^ Department of Clinical and Molecular Medicine Sant'Andrea Hospital Sapienza University of Rome Rome Italy; ^2^ Department of Neurosciences Mental Health & Sensory Organs (NESMOS) Sapienza University of Rome Rome Italy; ^3^ Department of Medical and Surgical Science University Magna Graecia Catanzaro Italy; ^4^ Department of Experimental Medicine Sapienza University of Rome Rome Italy

**Keywords:** COVID‐19, male fertility, mRNA, SARS‐CoV‐2, semen, vaccine

## Abstract

**Background:**

The effects of an mRNA COVID‐19 vaccine on spermatozoa parameters are not known. The aim of this study was to evaluate the effect of the BNT162b2 mRNA COVID‐19 vaccine on human semen, comparing spermatozoa parameters before and after vaccine inoculation.

**Materials and methods:**

In this single‐center prospective study, voluntary subjects who received mRNA vaccines from February to July 2021 were enrolled. The study population included male subjects aged between 18 and 45 years who completed the BNT162b2 mRNA COVID‐19 vaccine cycle. All subjects were evaluated before the first dose of vaccine (T0) and after 3 months (T1) with semen analysis and further analysis of seminal plasma, including colorimetric determination of reactive oxygen metabolites (d‐ROM test), electrolytes, and interleukin 6 (IL‐6) assessment by enzyme‐linked immunosorbent assay technology.

**Results:**

The experimental sample included 47 subjects (age: 29.3 ± 6.0 years, range 24–32; body mass index: 23.15 ± 2.5 kg/m^2^, range 19.2–28.0). All the subjects reported no systemic side effects. No significant differences were observed in any spermatozoa parameter between T0 and T1. A subanalysis was performed in oligoazoospermic and asthenozoospermic subjects, confirming the same results. Electrolyte analysis also showed no significant differences before and after vaccine inoculation. Finally, no significant differences were observed in T0, compared to T1 for the d‐ROM test and IL‐6.

**Discussion and conclusion:**

In this study, no significant differences in spermatozoa parameters before and after vaccine inoculations were found. Furthermore, oxidative stress analysis,, the activity of the cell membrane, and IL‐6, as a marker of inflammation, was not affected by the mRNA COVID‐19 vaccine. These results suggest that this vaccine is safe for male semen quality.

## 1 INTRODUCTION

The Coronavrus Disease 2019 (COVID‐19) Severe acute respiratory syndrome coronavirus 2 of the genus Betacoronavirus (SARS‐CoV‐2) virus spread into a global pandemic, which is responsible for over millions of deaths and economic suffering. Diabetes mellitus, chronic pulmonary obstructive diseases, and malignancies were the most important predictors of mortality in an age‐ and sex‐dependent manner.^1^

Two mRNA vaccines received use authorization from the European Medicines Agency (EMA) in December 2020.^2,^ ^3^ One of them, the mRNA vaccine BNT162b2 (Pfizer‐BioNTech), was first used in Italy. The release of non‐replicating RNA within the cell host allows for directing spike protein SARS‐CoV‐2 expression. The vaccine induces both a neutralizing antibody response and a cell‐mediated immune response to the antigen of the spike protein (“S”), which can induce protection against SARS‐CoV‐2 infection.^3^

Several studies reported that SARS‐CoV‐2 infection could be associated with an impairment in testicular function.^4–^ ^8^ However, few studies have been carried out to investigate the possible correlation between infection and spermatozoa parameter impairment.^5^

Xu et al. reported structural changes in testicular cells (Leydig and Sertoli cells as well as spermatogonia), which can affect the reproductive system; the analysis was conducted *post‐mortem* in patients with SARS coronavirus infection.^9^ In particular, vacuolation and cytoplasmic dilution of Sertoli cells, loss of integrity of the basement membrane, and seminiferous tubules and inflammatory cell infiltrate were observed. This inflammatory infiltrate seems to be related to the presence of a high viral load for which protein S is responsible.^9^

EMA published a data sheet that included potential adverse events of the COVID‐19 vaccine.^3^ Despite the few adverse events found in clinical trials, mainly characterized by short‐term, mild‐to‐moderate pain at the injection site, fatigue, and headache,^10^ very few data are available on the potential reproductive toxicity of the SARS‐CoV‐2 vaccine. In this regard, an animal study was performed in female mice; no significant alterations in reproductive function were observed following SARS‐CoV‐2 vaccination.^3^, ^11^ On the other hand, no studies were conducted to evaluate the reproductive function in males before the authorization of the vaccine.

In general, there are few studies concerning reproductive toxicity vaccine related; however, all studies are in agreement in stating that any alterations in spermatogenesis can occur mainly as a consequence of adverse reactions, such as an increase in body temperature above 38°C, and represent a transitory effect.^12,^ ^13^ On the other hand, Chaudhary et al. reported adverse reactions related to the use of vaccine excipients and/or adjuvants.^12^ Previously, Xu et al. studied the reproductive toxicity of adjuvants such as silver nanoparticles^14^ in rabies virus vaccines and highlighted that their small size can allow crossing through biological barriers such as the blood–testis barrier, inducing cytotoxicity (inflammatory state and a decrease in mitochondrial function with the production of reactive oxygen species [ROS] and an increase in cellular apoptosis and/or a downregulation of gene expression, which could induce cellular apoptosis). Moreover, cytotoxicity has been associated with electrolyte imbalance between the intracellular membrane and the extracellular membrane, which leads to an alteration of metabolic activities.^15^, ^16^

Furthermore, there is a lack of knowledge and understanding about newly developed mRNA‐based vaccines induced in the popular media the fear of a potential association between the SARS‐CoV‐2 vaccine and male infertility. The confusion generated in public opinion has brought to light widespread concern about any short‐term reactions but, above all, about the long‐term side effects of vaccines.

To date, two studies have been conducted to investigate the relationship between spermatozoa parameters and the BNT162b2 mRNA Covid‐19 vaccine.^17,^ ^18^ In both studies, the authors found no significant abnormality in any spermatozoa parameter, but some drawbacks limit the significance of these analyses, of which the most relevant was that progressive motility and morphology were not considered^17^ and that the analysis was performed approximativelyafter 1–2 months following their second dose.^18^

The aim of this study was to evaluate the effect of the BNT162b2 mRNA Covid‐19 vaccine on human semen, comparing spermatozoa parameters before and after vaccine inoculation. Furthermore, to exclude the negative effects of vaccines related to oxidative stress, cell membrane activity and inflammation, we also assessed reactive oxygen metabolites, electrolytes, and interleukin 6 (IL‐6) in seminal plasma.

## 2 MATERIALS AND METHODS

In this single‐center prospective study, voluntary subjects recruited from the administrative and clinical staff of the hospital who underwent mRNA vaccination from February to July 2021 were enrolled.

The study population included male subjects, Caucasian, aged between 18 and 45 years who completed the BNT162b2 mRNA COVID‐19 vaccine cycle (first and second doses after 1 month).

The exclusion criteria were (a) previous SARS‐CoV‐2 infection, (b) azoospermia, (c) use of medications and/or nutraceutics and/or surgery during the study's time frame, and (d) urinary tract infections, genetic diseases, neoplasms, or exposure to chemotherapy or radiotherapy treatments.

Age, body mass index (BMI) and any reaction to the first or second dose of the vaccine were recorded. Informed written consent was obtained from all the patients, and the study was performed according to the Declaration of Helsinki. The procedures were approved by a local ethics committee: “Sapienza” University of Rome Ethics Committee (Rif. CE 6280_2021).

### 2.1 Semen analysis

**FIGURE 1:**
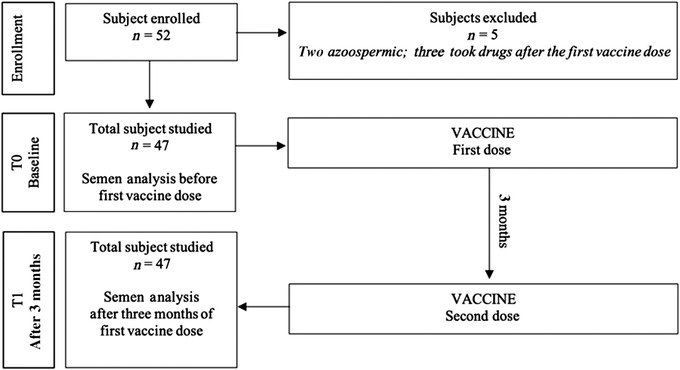
Diagram of the study

Semen analysis was performed before the first dose of vaccine (T0) and after 3 months (T1), which corresponds to 70 days from the second dose (Figure 1).

Seminal fluid of each participant was collected by masturbation after sexual abstinence between 2 and 7 days. All samples were allowed to liquefy at 37°C for 60 mins and were then assessed according to World Health Organization (WHO).^19^ Macroscopic examination (volume, appearance, pH, liquefaction, viscosity) and microscopic evaluation were carried out; in particular, morphology assessment was carried out after smear staining with the Bryan—Leishman method.^20^, ^21^ The following seminal parameters were evaluated: sperm concentration (10^6^/ml), total spermatozoa number (*n* × 106/ejaculate), total motility and progressive motility (%), morphology (% abnormal forms), and round cells (*n* x10^6^/ml). After semen analysis, all the samples were centrifuged for 10 min at 2000 x g with nucleofuge |24 d (diatech‐pharmacogenetics) and were subsequently used for the assay of determination of reactive oxygen metabolites (d‐ROMs), electrolytes, and IL‐6. The optical density for the IL‐6 and d‐ROM tests was evaluated with a READWELL TOUCH ROBONIK (enzyme‐linked immunosorbent assay [ELISA] plate analyzer‐Tech Medisystems).

### 2.2 Colorimetric d‐ROMs

In the d‐ROMs test, ROMs of the seminal plasma, in the presence of iron, are able to generate alkoxyl (R−O*) and peroxyl (R−OO*) radicals that are able to oxidize an alkyl‐substituted aromatic amine (that is solubilized in a chromogenic mixture), thus transforming them into a pink‐colored derivative. The colored derivative is photometrically quantified. When the reagents are at working temperature, a blank reagent and a calibrator with assigned values are prepared for each series of assays. This value is reported in the sticker on the control serum vial. All the solutions were delicately mixed, and after 1 min of incubation at 37°C, a photometric reading was performed by measuring their absorbance (at 505 or 546 nm) immediately and after 1, 2, and 3 min under the same operating conditions (37°C). Afterwards, the absorbance value of the reagent blank was subtracted from that of the calibrator and the samples. Finally, the results of the d‐ROM test are expressed as Carratelli Units (CARR U; Diacron International).

### 2.3 Electrolytes

The electrolytes were assessed by automated instrumentation. Architect cSystems A total of 300 μl of each sample was used for the assay. Sodium, potassium, and chlorine were quantified using ion‐selective electrodes (ICT Module Aeroset For Architect C16000, C8000, and Ci8200 Analyzers #09D2803; Abbott—01E4920‐BX—ICT Reference Solution Architect General Chemistry For Architect c16000 Analyzer). Calcium (Abbott—03L7921‐BX—Calcium Reagent Architect General Chemistry Calcium) was determined a colorimetric method using the arsenazo III and magnesium (Abbott‐03P6822‐EA‐REAGENT, MAGNESIUM) was obtained with the use of an enzymatic reaction in accordance with the manufacturer's instructions.

### 2.4 IL‐6

IL‐6 was assessed by ELISA technology, which allows the formation of the antibody‐antigen complex in one single step in 90 min. The procedure included seminal plasma and antibody mixing, incubation, washing, and adding the final substrate. All samples were analyzed by commercially available IL‐6 ELISA kits (Abcam ab178013) in accordance with the manufacturer's instructions.

### 2.5 Statistical analysis

All statistical analyses were performed using R version 4.0.3 (2020‐10‐10)—“Bunny‐Wunnies Freak Out” Copyright 2020 The R Foundation for Statistical Computing). The normality of the variables was assessed with the D'Agostino—Pearson normality test. The difference between the normal variables at baseline and time two was assessed by paired samples *t*‐test, while the difference between non‐parametric variables was evaluated with Wilcoxon's signed rank test. Correlation analyses were performed using Spearman rank correlation with corplot package. Data are reported as the mean ± SD or median and confidence interval (CI) of the median. *p* < 0.05 was considered statistically significant.

Considering a statistical power of 90% and an error *α* of 0.05, considering an average sperm concentration in healthy subjects aged between 18 and 45 years of 80 ± 20 × 10^6^/ml,^11^ and a non‐significant variation of this parameter ≥10% (70 ± 20 × 10^6^/ml), a minimum sample of 47 subjects was required.

## 3 RESULTS

A total of 52 subjects were enrolled. Of them, five subjects were excluded (two were azoospermic; three subjects took drugs after the first vaccine dose).

Thus, the final sample included 47 subjects (age: 29.3 ± 6.0 years, range 24—32; BMI: 23.15 ± 2.5 kg/m^2^, range 19.2—28.0).

A total of 5/47 (10.6%) subjects were moderate smokers, and 18/47 (38.3%) reported social alcohol intake. All the subjects reported no systemic side effects (fever and/or allergic reaction and/or lymphadenopathy).

No significant differences were observed in spermatozoa parameters in T0, compared to T1 (volume 4.0 ± 2.0 vs. 4.3 ± 1.9 ml, sperm concentration [10^6^/ml] 74.4 ± 25.9 vs. 72.2 ± 30.0 × 10^6^/ml, total spermatozoa number 272.6 ± 152.8 vs. 272.6 ± 152.8 [10^6^/ejaculate], total motility 46.9 ± 11.5 vs. 46.1 ± 10.6%, progressive motility 46.5 ± 12.2 vs. 45.3 ± 11.3%, abnormal forms 81.3 ± 6.7 vs. 79.5 ± 7.4%; *p* > 0.05; Table 1).

**TABLE 1:** Spermatozoa parameters before the first dose (T0) and after 3 months (T1) of injection BNT162b2 mRNA COVID‐19 vaccine

**Spermatozoa parameters**	**T0 (n.47)**	**T1 (n.47)**	***p*‐value**
Volume (ml) Mean ± SD	4.0 ± 2.0	4.3 ± 1.9	0.5
Concentration (*n* x 10^6^/ml) Mean ± SD	74.4 ± 25.9	72.2 ± 30.0	0.7
Total spermatozoa number (*n* x 10^6^/ejac) Mean ± SD	272.6 ± 152.8	294.5 ± 162.6	0.6
Total sperm motility (%) Mean ± SD	46.9 ± 11.5	46.1 ± 10.6	0.7
Progressive motility (%) Mean ± SD	46.5 ± 12.2	45.3 ± 11.3	0.7
Abnormal morphology form (%) Mean ± SD	81.3 ± 6.7	79.5 ± 7.4	0.3
Round cells (*n* x 10^6^/ml) Mean ± SD	0.6 ± 0.3	0.6 ± 0.4	1.0

A subanalysis was performed in oligoazoospermic and asthenozoospermic subjects (overall, 12/47, 25.5%). No significant differences were observed in the latter group at T0, compared to T1 (volume 4.6 ± 1.8 vs. 4.9 ± 1.5 ml, sperm concentration [10^6^ /ml] 56.5 ± 27.7 vs. 59.5 ± 29.4 10^6^/ml, total spermatozoa number [10^6^/ejaculate] 257.3 ± 148.0 vs. 293.3 ± 152.9, total sperm motility 33.4 ± 4.3 vs. 40.1 ± 10.6%, abnormal forms 85.0 ± 5.4 vs. 78.8 ± 9.5%; *p* > 0.05); however, an improving trend was observed.

Electrolyte analysis also showed no significant differences in T0, compared to T1 (calcium 18.3 ± 5.6 vs. 18.1 ± 5.3 mg/dl, chlorine 33.4 ± 7.4 vs. 32.8 ± 7.0 mmol/L, potassium 24.1 ± 8.5 vs. 23.9 ± 9.2 mmol/L, magnesium 7.8 ± 4.8 vs. 7.7 ± 5.1 mmol/L, sodium 82.5 ± 34.4 vs. 84.9 ± 26.7 mmol/L; *p* = NS; Table 2).

**TABLE 2:** Electrolytes, oxidative stress, and interleukin‐6 (IL‐6) in seminal plasma before the first dose (T0) and after 3 months (T1)

	**T0 (n.47)**	**T1 (n.47)**	***p‐*value**
Calcium (mg/dl) Mean ± SD	18.3 ± 5.6	18.1 ± 5.3	0.9
Chlorine (mmol/L) Mean ± SD	33.4 ± 7.4	32.8 ± 7.0	0.8
Potassium (mmol/L) Mean ± SD	24.1 ± 8.5	23.9 ± 9.2	0.9
Magnesium (mmol/L) Mean ± SD	7.8 ± 4.8	7.7 ± 5.1	1.0
Sodium (mmol/L) Mean ± SD	82.5 ± 34.3	84.9 ± 26.7	0.8
droms_UCARR/ml Median (95% confidence interval [CI])	596.6(446.05–719.16)	636.0(512.98–777.74)	0.6
IL‐6 (pg/ml) Median (95% CI)	29.0(23.74–36.00)	26.0(24.00–34.25)	0.9

Furthermore, colorimetric determination of ROMs revealed no differences in T0, compared to T1 (median 596.6 UCARR/ml [446.0–719.2 95% CI of median] vs. median 636.0 UCARR/ml [512.9–777.7 95% CI of median], *p* = 0.6; Table 2). Considering IL‐6, no significant differences were observed in T0, compared to T1 (median 29.0 pg/ml [23.7–36.0 95% CI of median] vs. median 26.00 pg/ml [24.00–34.25 95% CI of median; *p* = 0.9]; Table 2).

Finally, no significant correlations were observed between all spermatozoa parameters and electrolytes as well as d‐ROM test and IL‐6, with the exception of a weak inverse correlation between T0 sodium and T1 IL‐6 (*r* = −0.2).

## 4 DISCUSSION

Testicular damage and subsequent infertility have been hypothesized to be a consequence of COVID‐19 infection.^4^ Several studies have shown that SARS‐CoV‐2 testis localization can increase the forthcoming risk of spermatozoa production impairment and hypogonadism.^6^, ^7^ Similarly, low testosterone in the acute phase of the disease can increase the risk of worse outcomes.^8^

SARS‐CoV‐2 in seminal fluid has been studied by several authors,^21^, ^22^, ^23^, ^24^ and viral RNA in semen samples was not detected in all of the studies. However, partially in contrast with these results, Gacci et al. found one patient, out of 43, positive for SARS‐CoV‐2 RNA in semen.^25^ Furthermore, in a study conducted in China by Li et al., viral RNA was identified in the semen of infected patients; however, false positive results could be due to contamination with respiratory droplets in the semen containers.^26^ As a consequence, couples delayed planning their pregnancies for fear of infection.^27^, ^28^ Furthermore, because of the lack of studies conceived to evaluate reproductive function in humans before the authorization of this new type of vaccine, there was a spread of misinformation on social media stating that BNT162b2 vaccines could cause sterility.^27^

In this regard, even in the absence of specific studies, the Society for Male Reproduction and Urology and the Society for the Study of Male Reproduction recommend that the COVID‐19 vaccine should not be withheld from men desiring fertility who meet the criteria for vaccination and should be offered to men desiring fertility, similar to men not desiring fertility, when they meet the criteria for vaccination.^29^ The authors highlighted that abouty 16% of men experienced fever after the second dose, which can cause temporary declines in spermatozoa quality.^29^

To date, two studies have been conducted to investigate the relationship between spermatozoa parameters and the SARS‐CoV‐2 vaccine,^17,^ ^18^ both conducted after authorization. The authors found no significant abnormalities in any spermatozoa parameter, but the studies had some limitations. Gonzalez et al.^17^ did not consider progressive motility and morphology, while Lifshitz et al.^18^ compared semen analysis before and approximatively after 1–2 months following their first dose, which does not cover the complete spermatozoa maturation process from a testicular stem cell, which takes around 74 days.

According to these data, in the present study, we found no significant differences in sperm concentration, motility, and morphology when comparing values before and after vaccine inoculations. We also analyzed the effects of the vaccine in subjects with oligo/asthenozoospermia, and we observed the same results. Moreover, an improving trend in all spermatozoa parameters could be noted in this subgroup, particularly in motility. However, according to Gonzalez et al.,^17^ this improvement can be associated with the normal individual variation in spermatozoa parameters.

Furthermore, we also evaluated reactive oxygen metabolites, electrolytes, and IL‐6 in seminal plasma. Interestingly, we found no differences in the d‐ROM test, expression of oxidative stress‐ and free radical‐derived compounds. Recent evidence showed that oxidative stress could increase the affinity of SARS‐CoV and SARS‐CoV‐2 protein “S” for the angiotensin‐converting enzyme 2 receptor; this mechanism, therefore, could increase the severity of COVID‐19 infection.^30^ On the other hand, it is well known that oxidative stress could play an independent role in the etiology semen impairment by hindering the capacitation process and damaging spermatozoa membrane and DNA.^31^ In this regard, approximately 30%–80% of infertile men showed higher seminal ROS levels.^31^

The analysis of the electrolytes showed good cell membrane activity, which was stable after vaccine inoculation. In fact, an electrolytic balance must be present between the intracellular membrane (chlorine, calcium, magnesium, potassium) and extracellular space (sodium) for the cell to carry out its normal functions.^15^ Defective expression of each electrolyte has been described in several studies on the basis of spermatogenic dysfunctions; in this regard, electrolyte analyses of human seminal fluid have been previously performed.^16^, ^32^, ^33^ Specifically, Nag et al. observed a significantly lower Na concentration in asthenozoospermic subjects than in normospermic subjects.^32^ Furthermore, Bondani et al. showed that K levels were higher in oligoasthenospermic subjects than in normospermic subjects.^16^

Considering IL‐6 dosage, a marker of inflammation, the analysis revealed no significant differences before and after vaccination. It is known that the IL‐6 concentration in seminal plasma is higher in infertile men and correlates with reduced motility.^34^ Furthermore, high IL‐6 levels in blood seem to be associated with adverse clinical outcomes in patients with COVID‐19.^35^

Finally, we could speculate that the weak inverse correlation between T0 sodium and T1 IL‐6 indicates an inverse correlation between better membrane activity^32^ and lower inflammation.

The main limitations of this study are the small number of oligozoospermic individuals, the group of subjects likely most susceptible to worsening semen parameters, and that the study group came from a specific population and were not random people.

## 5 CONCLUSION

In conclusion, no significant differences were observed between semen parameters, oxidative stress analysis, or IL‐6 as a marker of inflammation and electrolyte function before and after the BNT162b2 mRNA COVID‐19 vaccine. Our results suggest that this kind of COVID‐19 vaccine is safe for male semen quality.

## CONFLICT OF INTEREST

The authors have no conflicts of interest to disclose.

## AUTHOR CONTRIBUTIONS

Soraya Olana, Rossella Mazzilli, and Antongiulio Faggiano conceived and designed the study. Soraya Olana, Rossella Mazzilli, Virginia Zamponi, and Maria Grazia Tarsitano acquired the data. Soraya Olana, Rossella Mazzilli, Gerardo Salerno, and Virginia Zamponi analyzed and interpreted the data. Soraya Olana and Rossella Mazzilli drafted the article. Rossella Mazzilli, Donatella Paoli, and Antongiulio Faggiano critically revised the manuscript for important intellectual content. All authors approved the final version to be submitted and declare agreement to be accountable for all aspects of the work in ensuring that questions related to the accuracy or integrity of any part of the work are appropriately investigated and resolved.

## References

[1] Corona G. , Pizzocaro A , Vena W , et al. Diabetes is most important cause for mortality in COVID‐19 hospitalized patients: systematic review and meta‐analysis. Rev Endocr Metab Disord. 2021;22(2):275–296. 10.1007/s11154-021-09630-8 33616801 PMC7899074

[2] Szilagyi PGTK , Thomas K , Shah MD , et al. National trends in the US public's likelihood of getting a COVID‐19 vaccine: April 1 to December 8, 2020. JAMA. 2020;325(4):396–398. 10.1001/jama.2020.26419 PMC7772743 33372943

[3] https://www.ema.europa.eu/en/medicines/human/EPAR/comirnaty

[4] Corona G , Baldi E , Isidori AM , et al. SARS CoV 2 infection, male fertility and sperm cryopreservation: a position statement of the Italian Society of Andrology and Sexual Medicine (SIAMS) (Società Italiana di Andrologia e Medicina della Sessualità). J Endocrinol Invest. 2020;43(8):1153–1157. 32462316 10.1007/s40618-020-01290-w PMC7252417

[5] Aitken RJ . COVID‐19 and human spermatozoa–potential risks for infertility and sexual transmission? Andrology. 2021;9(1):48–52. 32649023 10.1111/andr.12859 PMC7404878

[6] Salonia A. , Pontillo S. , Capogrosso P. , et al. Testosterone in males with COVID‐19: a 7‐mounth cohort study. Andrology. 2022;10(1):34–41. 10.1111/andr.13097 34409772 PMC8444879

[7] Cinisliouglu AM. , Cinisliouglu N. , Demirdogen SO. , et al. The relationship of serum testosterone levels with the clinical course and prognosis of COVID‐19 disease in male patients: a prospective study. Andrology. 2022;10(1):24–33. 10.1111/andr.13081 34288536 PMC8444851

[8] Salonia A. , Corona G. , Giwercman A. , et al. SARS‐CoV‐2, testosterone and frailty in males (PROTEGGIMI): a multidimensional research project. Andrology. 2021;9(1):19–22. 10.1111/andr.12811 32369678

[9] Xu J , Qi L , Chi X , et al. Orchitis: a complication of severe acute respiratory syndrome (SARS). Biol Reprod. 2006;74(2):410–416. 16237152 10.1095/biolreprod.105.044776 PMC7109827

[10] Polack FP , Thomas SJ , Kitchin N , et al. C4591001 clinical trial group. safety and efficacy of the BNT162b2 mRNA Covid‐19 vaccine. N Engl J Med. 2020;383(27):2603–2615. 33301246 10.1056/NEJMoa2034577 PMC7745181

[11] https://www.cdc.gov/coronavirus/2019‐ncov/vaccines/recommendations/pregnancy.html

[12] Chaudhary JK , Yadav R , Chaudhary PK , et al. Insights into COVID‐19 vaccine development based on immunogenic structural proteins of SARS‐CoV‐2, host immune responses, and herd immunity. Cells. 2021;10(11):2949. PMID 34831172. 10.3390/cells10112949 34831172 PMC8616290

[13] Samplaski MK , Nangia AK . Adverse effects of common medications on male fertility. Nat Rev Urol. 2015;12:401–413. 26101108 10.1038/nrurol.2015.145

[14] Xu L . Wang YY , Huang J , et al. Silver nanoparticles: synthesis, medical applications and biosafety. Theranostics. 2020;10(20):8996–9031. PMID: 32802176 10.7150/thno.45413 32802176 PMC7415816

[15] Brown SG , Publicover SJ , Barratt CLR , da Silva SJM . Human sperm ion channel (dys)function: implications for fertilization. Hum Reprod Update. 2019;25(6):758–776. 31665287 10.1093/humupd/dmz032 PMC6847974

[16] Bondani A , Aspeitia E , Aznar R , Gómez‐Arzápalo E , Pascual C , Giner J . Correlation between sperm motility and electrolyte composition of seminal fluid in normal and infertile men. Fertil Steril. 1973;24(2):150–154. 4688103 10.1016/s0015-0282(16)39498-5

[17] Gonzalez DC , Nassau DE , Khodamoradi K , et al. Sperm parameters before and after COVID‐19 mRNA vaccination. JAMA 2021;326(3):273–274. 34137808 10.1001/jama.2021.9976 PMC8293015

[18] Lifshitz D , Haas J , Lebovitz O , Raviv G , Orvieto R , Aizer A . Does mRNA SARS‐CoV‐2 vaccine detrimentally affect male fertility, as reflected by semen analysis? Reprod Biomed Online. 2021;S1472‐6483(21):00480–6. 10.1016/j.rbmo.2021.09.021 PMC8489287 34815157

[19] World Health Organization. *Laboratory Manual for the Examination and Processing of Human Semen* . 5th ed. Cambridge University Press; 2010.

[20] Belsey MA , Eliasson R , Gallegos AJ , et al. Laboratory Manual for the Examination of Human Semen and Semen‐Cervical Mucus Interaction. Press Concern, cop; 1980.

[21] Williams MA , Wick A , Smith DC . The influence of staining procedure on differential round cell analysis in stained smears of human semen. Biotech Histochem. 1996;71(3):118–122. 8724436 10.3109/10520299609117147

[22] Paoli D , Pallotti F , Turriziani O , et al. SARS‐CoV‐2 presence in seminal fluid: myth or reality. Andrology. 2021;9(1):23–26. 32453494 10.1111/andr.12825 PMC7283802

[23] Song C , Wang Y , Li W , et al. Absence of 2019 novel coronavirus in semen and testes of COVID‐19 patients†. Biol Reprod. 2020;103(1):4–6. 32297920 10.1093/biolre/ioaa050 PMC7184456

[24] Pan F , Xiao X , Guo J , et al. No evidence of severe acute respiratory syndrome‐coronavirus 2 in semen of males recovering from coronavirus disease 2019. Fertil Steril. 2020;113(6):1135–1139. 32482249 10.1016/j.fertnstert.2020.04.024 PMC7164916

[25] Gacci M , Coppi M , Baldi E , et al. Semen impairment and occurrence of SARS‐CoV‐2 virus in semen after recovery from COVID‐19. Hum Reprod. 2021;36(6):1520–1529. 33522572 10.1093/humrep/deab026 PMC7953947

[26] Li D , Jin M , Bao P , et al. Clinical characteristics and results of semen tests among men with coronavirus disease 2019. JAMA Netw Open. 2020;3:e208292. 32379329 10.1001/jamanetworkopen.2020.8292 PMC7206502

[27] Allahbadia G . Will procreation ever be the same after COVID‐19? J Obstet Gynaecoll India. 2021;71(1):1–6. 10.1007/s13224-021-01536-4 PMC8403817 34483511

[28] Mazzilli R , Zamponi V , Faggiano A . Letter to the editor: how the COVID‐19 pandemic has changed outpatient diagnosis in the andrological setting. J Endocrinol Invest. 2021; 10:1–2. 10.1007/s40618-021-01673-7 PMC8429890 34506035

[29] Joint statement regarding COVID‐19 vaccine in men desiring fertility from the Society for Male Reproduction and Urology (SMRU) and the Society for the Study of Male Reproduction (SSMR) 2021; https://www.asrm.org/news‐and‐publications/covid‐19/statements/joint‐statement‐regarding‐covid‐19‐vaccine‐in‐men‐desiring‐fertility‐from‐the‐society‐for‐male‐reproduction‐and‐urology‐smru‐and‐the‐society‐for‐the‐study‐of‐male‐reproduction‐ssmr/

[30] Hati S , Bhattacharyya S . Impact of thiol‐disulfide balance on the binding of Covid‐19 spike protein with angiotensin‐converting enzyme 2 receptor. ACS Omega. 2020;5(26):16292–16298. 10.1021/acsomega.0c02125 PMC7346263 32656452

[31] Agarwal A , Parekh N , Panner Selvam MK , et al. Male Oxidative Stress Infertility (MOSI): proposed terminology and clinical practice guidelines for management of idiopathic male infertility. World J Mens Health. 2019;37(3):296–312. 31081299 10.5534/wjmh.190055 PMC6704307

[32] Nag A , Chaudhuri N . Electrolyte content of human seminal fluid at different states of fertility. Indian J Exp Biol. 1978;16(9):954–956. 721152

[33] Kavanagh JP . Sodium, potassium, calcium, magnesium, zinc, citrate and chloride content of human prostatic and seminal fluid. J Reprod Fertil. 1985;75(1):35–41. 4032375 10.1530/jrf.0.0750035

[34] La Vignera S , Condorelli RA , Vicari E et al. Microbiological investigation in male infertility: a practical overview. J Med Microbiol. 2014;63(1):1–14. 24072761 10.1099/jmm.0.062968-0

[35] Coomes EA , Haghbayan H . Interleukin‐6 in Covid‐19: a systematic review and meta‐analysis. Rev Med Virol. 2020;30(6):1–9. 10.1002/rmv.2141 PMC7460877 32845568

